# Beyond AMR and virulence databases: genome wide associations of closely related *Vibrio alginolyticus* and emerging *Vibrio diabolicus* provide framework for identifying novel genetic markers

**DOI:** 10.3389/fmicb.2026.1796882

**Published:** 2026-07-09

**Authors:** Peter J. Sebastian, Cory Schlesener, Barbara A. Byrne, Melissa Miller, Bart C. Weimer, Christine K. Johnson

**Affiliations:** 1 Institute for Pandemic Insights, Weill School of Veterinary Medicine, University of California, Davis, Davis, CA, United States; 2 Karen C. Drayer Wildlife Health Center, One Health Institute, Weill School of Veterinary Medicine, University of California, Davis, Davis, CA, United States; 3 100K Pathogen Genome Project, Department of Population Health and Reproduction, Weill School of Veterinary Medicine, University of California, Davis, Davis, CA, United States; 4 Department of Pathology, Microbiology and Immunology, Weill School of Veterinary Medicine, University of California, Davis, Davis, CA, United States; 5 Marine Wildlife Veterinary Care and Research Center, California Department of Fish and Wildlife, Santa Cruz, CA, United States

**Keywords:** antimicrobial resistance, emerging pathogen, genomic epidemiology, GWAS – genome wide association study, *Vibrio alginolyticus*, *Vibrio diabolicus*, *Vibriosis*, virulence

## Abstract

*Vibrio alginolyticus* is a frequently implicated species for vibriosis in humans and diverse wildlife, but it has previously been difficult to identify from the closely related and emerging Vibrio *diabolicus*. Comparisons of both species, including antimicrobial resistance (AMR) and virulence characterizations, are scarce and impeded by intraspecies diversity, minimal genomes, discordant classification methods, and gene databases with limited utility to understudied species. The species identities of 3,442 public domain genomes (SRA files) within the Harveyi clade were re-evaluated using genomic methods. Public genomes identified as *V. diabolicus* and *V. alginolyticus* were combined with previously published genomes isolated from humans, sea otters (*Enydra lutris*), or coastal environments (*V. diabolicus n* = 88, *V. alginolyticus n* = 163, *Vibrio parahaemolyticus n* = 287) for pangenome-wide association studies to identify species-specific gene clusters (95% identification threshold). Additional genome wide associations with isolation source (humans versus sea otters) were investigated, including AMR and virulence related gene clusters. Genomic reclassification identified 29 of 150 misclassified public domain *V. alginolyticus* genomes, including 26 reclassified as *V. diabolicus*. In total, 28 previously misclassified *V. diabolicus* genomes (*n* = 37 total) were identified, including 10 human-derived strains. GWAS identified 643 and 477 gene clusters specific to *V. alginolyticus* and *V. diabolicus*, respectively, while some multilocus sequencing analysis (MLSA) gene clusters were non-specific. Gene clusters (*n* = 109) associated with either *V. alginolyticus* isolated from humans or sea otters were identified including one annotated to a multidrug resistance gene (mdtk_1). No *V. diabolicus* gene clusters were associated with host species after multiple comparison correction, although pre-correction associations related to antimicrobial resistance were detected (cat_1, ampC). The genomic methods of classification presented provide accurate species identification for *V. diabolicus* and *V. alginolyticus* beyond current MLSA/MLST schemes, although target species-specific genes were identified that may be useful for improved future schemes. While limited sample size of *V. diabolicus* hampered the ability to detect host associated markers, the GWAS approach employed provide a reusable framework for discovering insights into host adaptation and prioritizing target genes for future functional AMR and virulence validation experiments in both species.

## 1 Introduction

The *Vibrionaceae* family is composed of over 190 species and 51 distinct clades, many of which are known to cause disease in marine wildlife or humans (Jiang et al., 2022). The causative agents of vibriosis, or non-cholera *Vibrio* spp. infections, are the leading public health threat from consumption of raw and undercooked seafood and recreational use of coastal waters (Baker-Austin et al., 2018). Some of the most frequently implicated *Vibrio* spp. derive from the Harveyi clade, including established pathogens of humans, seafood, and marine mammals: *Vibrio parahaemolyticus, Vibrio alginolyticus, and Vibrio harveyi* (Miller et al., 2020; Mougin et al., 2020; Culot et al., 2021). Harveyi clade species outbreaks are also linked to environmental factors including temperature. During low temperatures *Vibrio* spp. can enter a viable but non-culturable state and can quickly spread during favorable conditions (Baker-Austin et al., 2018; Yoon and Lee, 2020). Climate changes such as rising sea surface temperatures and more frequent marine heatwaves have already enhanced *Vibrio* spp. expansion and will likely facilitate *Vibrio* spp. infections in humans and marine mammals in the coming decades (Goertz et al., 2013; Baker-Austin et al., 2017; Cheung and Frölicher, 2020; Gulland et al., 2022).

As the risk of vibriosis increases over time, accurate taxonomic classification of the Harveyi clade will be increasingly critical to separate pathogenic and non-pathogenic species in humans, aquaculture, and wildlife. Most Harveyi clade classification schemes lack the resolution to identify isolates accurately and consistently at the species level. Classification of the Harveyi clade is complicated by under-sampling of emerging species, high intraspecies genomic diversity, and discordance between species identification methods (Culot et al., 2021; Song et al., 2021). One such under-sampled and emerging species is *Vibrio diabolicus*, whose pathogenicity and antimicrobial resistance to humans and marine wildlife is poorly studied (Song et al., 2021; Sebastian et al., 2025, 2026).

The *first* characterized *V. diabolicus* was isolated from a deep-sea hydrothermal vent annelid but has since been isolated from at least two humans, vertebrate and invertebrate seafood including a diseased grouper, and from diverse marine environments, with a global distribution (Raguénès et al., 1997; Dias, 2020; Song et al., 2021). While *V. diabolicus* is under-studied and under-sequenced in comparison to closely related *V. alginolyticus* and *V. parahaemolyticus*, recent sampling efforts suggest that it is ubiquitous in coastal environments and is a putative human and marine animal pathogen (Dias, 2020; Pérez-Duque et al., 2021; Sebastian et al., 2025, 2026). Whole genome sequencing of *Vibrio* spp., comparing genomes from multiple isolation sources, uncovered *V. diabolicus* isolates from northern (*kenyoni*) and southern (*nereis*) sea otters (*Enhydra lutris*) which were clinically misidentified (Sebastian et al., 2025, 2026).

Taxonomic classification of *V. diabolicus* has been difficult in part due to low resolution identification methods such as Matrix Assisted Laser Desorption Ionization-Time of Flight (MALDI-ToF), and Multi-Locus Sequence Analysis (MLSA) schemes that fail to accurately separate *V. alginolyticus* and *V. diabolicus* (Rahman et al., 2014; Culot et al., 2021; Sebastian et al., 2025). MLSA schemes may require frequent updates when emerging species are added because they rely on universal primers of “housekeeping” genes present in all species in the clade (Sawabe et al., 2013; Culot et al., 2021; Jiang et al., 2022). The limited set of gene markers used for MLSA may also not adequately account for emergent species and intra-species strain types. A higher resolution genomic comparison discovered that multiple genomes labeled as *V. alginolyticus* should be reclassified as *V. diabolicus*, and that the previously separate species *Vibrio antiquarius* is a synonym for *V. diabolicus* (Turner et al., 2018). Adding to the taxonomic confusion, the recently discovered *V. chemaguriensis*, which has not been validated by the International Code of Nomenclature of Bacteria, is most likely a synonym for *V. diabolicus* based on the most recently proposed *Vibrio* taxonomy (Ghosh and Bhadury, 2019; Schoch et al., 2020; Jiang et al., 2022).

Based on the frequent changes in Harveyi taxonomy that have impacted the classification of *V. diabolicus* within the last decade (Sawabe et al., 2013; Urbanczyk et al., 2013; Turner et al., 2018; Jiang et al., 2022), we hypothesized that public domain genomes would include misidentified *V. diabolicus* isolates. Our first aim was to re-classify publicly available genomes and highlight the advantages of genome-wide methods for species identification and strain relatedness compared to limited marker sequencing methods like MLSA and Multi-locus Sequence Typing (MLST) for differentiating *V. diabolicus* from closely related species.

Our previous investigations utilized a One Health approach to interrogate a large set of genomes of *V. parahaemolyticus, V. alginolyticus*, and *V. diabolicus* isolated from sea otters in Alaska and California along with corresponding isolates from bivalves and the nearshore marine environment to characterize antimicrobial resistance genes and virulence factors (Sebastian et al., 2025, 2026). The sea otter genomes provided a unique insight into the dynamics of *Vibrio* spp. infections in nearshore marine environments, given their potential for *Vibrio* spp. infections, the potential need for antimicrobial therapies, and the role of sea otters as sentinels of coastal environment health (Jessup et al., 2004, 2007; Brownstein et al., 2011; Miller et al., 2020; Burek Huntington et al., 2021). However, limitations in available gene databases utilized including the Comprehensive Antibiotic Resistance Database (Jia et al., 2017; Alcock et al., 2022) and Virulence Factor Database (Chen et al., 2016; Liu et al., 2022) rely heavily on known genes and mechanisms from better characterized *Vibrio* species, primarily *V. parahaemolyticus*.

Genome-wide association studies (GWAS) have emerged in the last 15–20 years as bioinformatics tools to identify novel genes associated with a particular species, phenotype, or genotype. Applications of GWAS have included attempts to identify novel genes associated with host or niche-adaptations as well as antimicrobial resistant and virulent phenotypes that may aid in identifying gene targets for molecular mechanistic experiments (Creasy-Marrazzo et al., 2022; Yang et al., 2022; Tiwari et al., 2023). Given the current challenges in typing and species identification of closely related *V. alginolyticus, V. diabolicus*, and *V. parahaemolyticus* using MLSA/MLST schemes, our second aim was to apply GWAS to filter species-specific genes as additional putative markers for future clinical species classification efforts. Finally, we aimed to illustrate applications of GWAS through identification of *V. alginolyticus* and *V. diabolicus* genes associated with host-specific niche adaptation to either sea otters or human hosts, including impacts that host tropism may have on antimicrobial resistance and virulence in emerging pathogenic species.

## 2 Methods

### 2.1 Sample collection

Sample collection and processing of *Vibrio* spp. isolates are previously described (Sebastian, 2023; Sebastian et al., 2025, 2026). Briefly, *n* = 268 *Vibrio* spp. isolates (55 *V. alginolyticus*, 52 *V. diabolicus*, and 161 *V. parahaemolyticus*) stored at −80°C at the University of California, Davis - Veterinary Medical Teaching Hospital were collected between 2000 and 2019 in Alaska, California, and Washington from various sources. Isolates were collected primarily across the central coast of California from avian feces (*n* = 2) (Siembieda et al., 2011; Oates et al., 2012), live-sampled and necropsied southern sea otters (*n* = 165) (Miller et al., 2010; Brownstein et al., 2011; Oates et al., 2012; Kreuder et al., 2013), bivalves (*n* = 19) (Miller et al., 2006), and environmental samples (water, sediment, algae, seagrass, and kelp swabs) from a June 2019 investigation of *Vibrio* spp. in Elkhorn Slough, California (*n* = 47) (Sebastian et al., 2025). Additional sampling included live and dead northern sea otters in Alaska from 2004 to 2015 (*n* = 32) (Goertz et al., 2013; Burek Huntington et al., 2021), and water samples from July 2019 off the coast of Protection Island, Washington (*n* = 3).

### 2.2 Whole genome sequencing and genome assembly

Isolates were plated on 5% sheep blood agar (Biological Media Services, University of California, Davis; Hardy Diagnostics, Santa Maria, CA) at 37°C with 5% CO_2_ for 24 h. DNA was extracted with either the QiaAMP UCP Pathogen extraction kit with mechanical pre-lysis using small pathogen lysis tubes (Qiagen, Hilden, Germany) or the Wizard genomic DNA purification kit (Promega Corporation, Madison, WI) followed by genomic DNA quality and yield assessment with the 2200 TapeStation and genomic DNA ScreenTape (Agilent Technologies, Santa Clara, CA) (Kong et al., 2013). Whole genome sequencing of *V. alginolyticus, V. diabolicus*, and *V. parahaemolyticus* isolates was performed as part of the 100K Pathogen Genome Project managed by the Weimer laboratory at the University of California, Davis (http://www.genomes4health.org/) (Kong et al., 2013, 2017; Lüdeke et al., 2015; Chen et al., 2017; Weimer, 2017). Library construction was performed with 1 μg fragmented gDNA using the HTP library preparation kit (Kapa Biosystems, Wilmington, MA) followed by WGS performed on Illumina HiSeq XTEN with PE 150 plus index read (Illumina, San Diego, CA) (Miller et al., 2015; Lawton et al., 2018). Trimming of adapters or Illumina standards was performed using Trimmomatic (v0.39) (Bolger et al., 2014) and quality of sequencing reads was assessed using FastQC (v0.11.9) (Andrews, 2010) before assembly using Shovill (v1.0.4) (Seemann, 2020). The quality of assembled genomes was assessed using FastQC and CheckM (v1.1.2) (Parks et al., 2015) to ensure >90% completeness, < 5% contamination, < 300 contigs, and >20 × genome coverage. Gene annotation was performed using Prokka (v1.14.6) (Seemann, 2014) and genomic species identification was determined using Kraken2 (v2.0.8) (Wood et al., 2019) with a RefSeq microbial genomes database (downloaded May 5, 2021) and Bracken (v2.6.1) (Lu et al., 2017).

The NCBI genome browser was screened on May 18th, 2022 for all available whole genome sequence SRA files with submitted species names within the Harveyi clade using the following search terms: {txid717610[Organism:exp] AND “wgs”[Strategy] AND “illumina”[Platform] AND “paired”[Layout]}. Samples with inconsistent file formats or lack of SRA availability were excluded. A total of 3,442 SRA files were downloaded including those submitted to NCBI as *V. parahaemolyticus* (*n* = 3,147), *V. alginolyticus* (*n* = 150), and *V. diabolicus* (*n* = 10; Supplemental Data 1). Public domain genomes were re-assembled from raw sequencing reads following the same genome assembly pipeline. An alluvial plot was constructed to compare the species classification submitted on NCBI with the re-assessed genomic species classification across Harveyi clade species using the ggplot2 R (v4.0.3) package.

Public domain genomes identified as *V. alginolyticus* or *V. diabolicus* through the Kraken2/Bracken pipeline were assessed with the same quality control metrics, except a stricter cut-off for genome coverage estimate (30 × ) for *V. alginolyticus* than *V. diabolicus* (20 × ) given the limited number of available *V. diabolicus*, before integration into downstream genome and pangenome analyses with previously published *V. parahaemolyticus* genomes (*n* = 126; 68 human, 58 oyster) (Miller et al., 2021) and genomes from the included sample collections for further downstream analysis (Supplemental Data 2). Metadata regarding isolation source was unavailable for genomes that were part of Pulsenet, although the “isolation type” listed for these strains was “clinical.” Genomes from Pulsenet were assumed to be isolated from humans based on the GenomeTrakr definition of clinical isolation type and the broader project description which states that isolates were primarily of human origin (Tolar et al., 2019). The seven-loci MLST scheme for *V. parahaemolyticus* typing (González-Escalona et al., 2008) was applied to all genomes using the bioconda mlst program (v2.23.0; T. Seemann, https://github.com/tseemann/mlst).

### 2.3 Genome and pangenome analyses

A k-mer based (31 k-mer scaled to 1,000 k-mers/Mbp) all-against-all MinHash sketch was generated using Sourmash (v3.2.3) to confirm appropriate clustering of genomes by their genomic species classifications (Brown and Irber, 2016; Bandoy and Weimer, 2020). Genomes that shared a Jaccard Similarity Index score of 0.9 or higher were considered near identical to identical. A core pangenome analysis was performed using Roary (v3.12.0) using the default 95% or higher identification threshold for unique gene clusters (Page et al., 2015). The core gene phylogeny and pangenome heat map were visualized online using Phandango (v1.3.0) (Hadfield et al., 2018). Genes clusters sharing the same Prokka annotation as the seven loci of the *V. parahaemolyticus* MLST scheme (González-Escalona et al., 2008), as well as the four loci *Vibrio* spp. MLSA scheme (Rahman et al., 2014) and the eight loci *Vibrio* spp. MLSA scheme (Sawabe et al., 2013), were compiled and visualized with the core gene phylogeny online using Phandango to assess their utility as MLSA markers.

Pangenome-association studies (pan-GWAS) were performed using Scoary (v.1.6.16) (Brynildsrud et al., 2016), which calculated a series of Fisher's exact tests, to quickly filter gene clusters from Roary that were highly associated with either *V. alginolyticus* (compared to *V. diabolicus* and *V. parahaemolyticus* combined) or *V. diabolicus* (compared to *V. alginolyticus* and *V. parahaemolyticus* combined). Shortened lists of gene clusters were filtered to those with at least 98% frequency in the target species and present in less than 5% of the remaining two species. The filtering was used to detect gene clusters that were core or near core to either species despite sample size differences that might otherwise bias partial species associations. Scoary outputs were parsed through eggnog-mapper (v2.1.6) to improve the functional annotation of significant gene clusters beyond Prokka annotations (Cantalapiedra et al., 2021).

Evaluation of phylogenetic clustering of *V. alginolyticus* and *V. diabolicus* isolates by isolation source (otter vs. human) was performed using distance-matrix–based multivariate statistical functions PERMDISP (test for homogeneity of multivariate dispersion, permutations = 10,000), PERMANOVA (permutational multivariate analysis of variance, permutations = 10,000), and ANOSIM (analysis of similarities, permutations= 10,000, test= “median,” method = “eigh”) in Scikit-Bio (version 0.6.3) with the “skbio.stats.distance” module (Aton et al., 2026). The statistical functions used a genomic similarity matrix generated by Sourmash (described above) converted to a distance matrix as 1 – Jaccard index. PERMDISP identified no dispersion for either *V. alginolyticus* (*F* = 2.82, *p* = 0.09) or *V. diabolicus* (*F* = 0.54, *p* = 0.97). PERMANOVA detected a significant difference in cluster distributions between isolation-source groups for *V. alginolyticus* (pseudo-*F* = 1.95, *p* < 0.001) but no significant differences for *V. diabolicus* (pseudo-*F* = 1.17, *p* = 0.11). There was significant but weak grouping of genomes by isolation source for both *V. alginolyticus* (*R* = 0.14, *p* = 0.0001) and *V. diabolicus* (*R* = 0.19, *p* = 0.015), suggestive of a weak benefit in population structure adjustment.

Additional GWAS comparisons within *V. alginolyticus* or *V. diabolicus* were performed to identify gene clusters (Roary) associated with either human or sea otter sourced genomes using PySeer (v.1.3.12) (Lees et al., 2018). PySeer was chosen over Scoary for intraspecies comparisons to account for population structure biases. The converted distance matrix was used as a distance parameter using a fixed effects SEER model for population structure adjustment. Features were filtered by allele frequencies between 10% and 90% and likelihood ratio test (lrt) *p*-values were assessed for significance after false discovery rate (FDR) adjustment. FDR adjustment was performed using SciPy (v1.16.2) and genomic features were considered significantly associated based on Benjamini–Hochberg adjusted lrt *p*-values < 0.05 (Virtanen et al., 2020). Previously published minimum inhibitory concentrations for antimicrobials in a subset of *V. alginolyticus* (*n* = 35), *V. diabolicus* (*n* = 18), and *V. parahaemolyticus* (*n* = 60) isolates from sea otters were added to Supplemental Data 2 (Sebastian et al., 2025).

## 3 Results

### 3.1 Reported vs. genomic species identification

**Figure 1:**
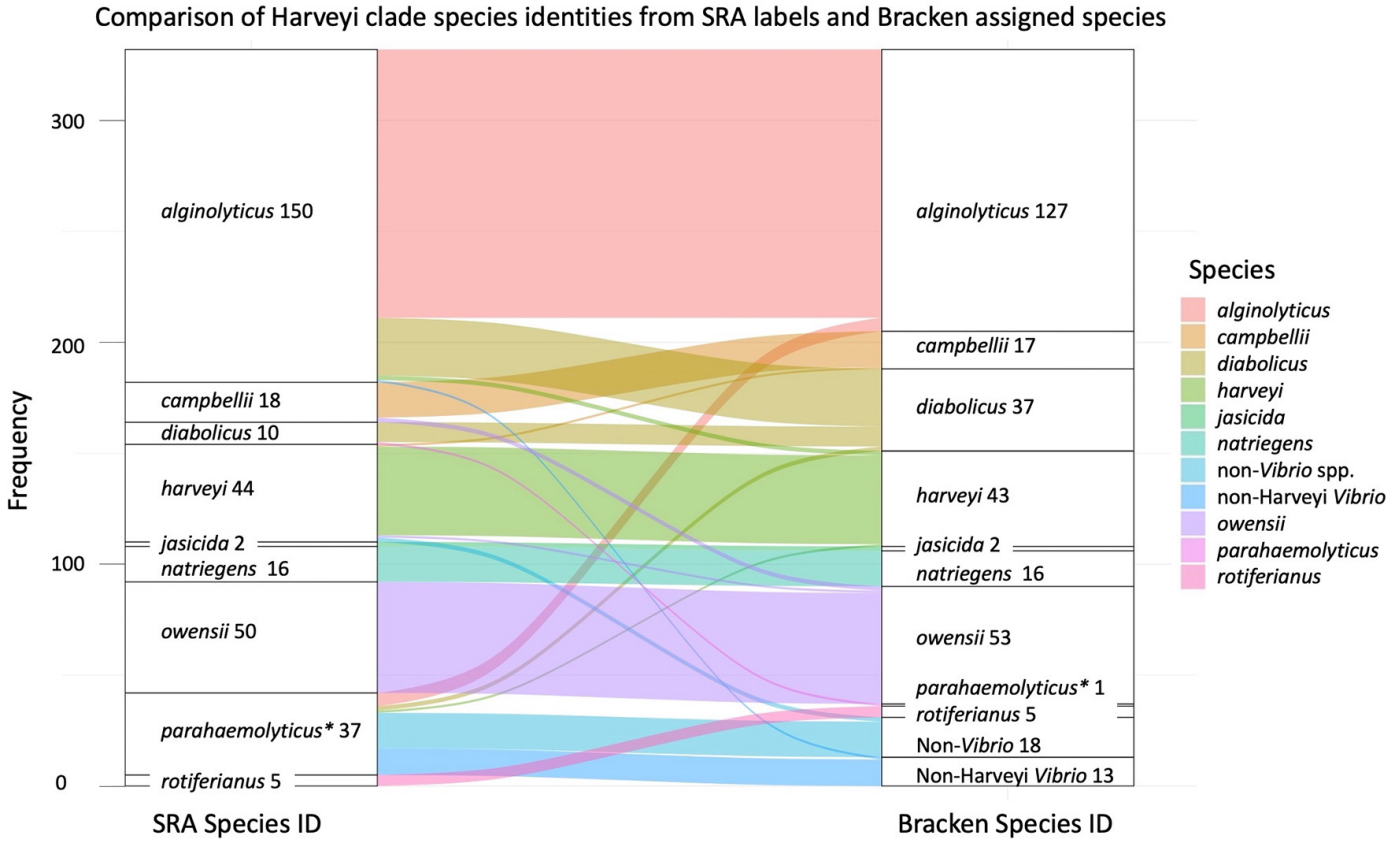
Alluvial diagram comparison between the reported species identities of 332 out of 3,442 NCBI uploaded whole genome sequence raw read files and the determined genomic species identities of *Harveyi clade Vibrio* species. *The 3,110 concordant *V. parahaemolyticus* isolates were not included in the flow chart. The most frequent misclassification involved *V. diabolicus* genomes reported on NCBI as *V. alginolyticus*.

Comparison of the reported species identities of 3,442 publicly available genomes from the Harveyi clade with genomically determined species identities revealed that 73 genomes were incorrectly classified (Figure 1; Supplemental Data 1). *Vibrio diabolicus* genomes were underreported as 28 out of 37 genomes that we confirmed as *V. diabolicus* had been incorrectly classified comprised of 26 and two misclassified as *V. alginolyticus* and *V. parahaemolyticus*, respectively. The accuracy of reported species identification was lowest for isolates labeled as *V. alginolyticus* (80.7%, (95% C.I. 73.4–86.7); 121 of 150).

Genomes reported as *Vibrio jasicida* (*n* = 2), *Vibrio natriegens* (*n* = 16), *Vibrio owensii* (*n* = 50), and *Vibrio rotiferianus* (*n* = 5) were all correctly identified. Genomes reported as *V. parahaemolyticus* were largely correctly identified (98.8% (98.4–99.2); 3,110 of 3,147) as were the remaining Harveyi clade species: *Vibrio campbelli* (16 of 18), *Vibrio diabolicus* (9 of 10), and *Vibrio harveyii* (40 of 44) collectively (90.3% (81.0–96.0); 65 of 72). Data entry errors were presumed to be minimal although mismatched genomes with identities either outside of the Harveyi clade (*n* = 18) or completely outside of the *Vibrio* genus (*n* = 13) were identified.

### 3.2 Genomic comparison between *V. alginolyticus, V. diabolicus*, and *V. parahaemolyticus*

**Figure 2:**
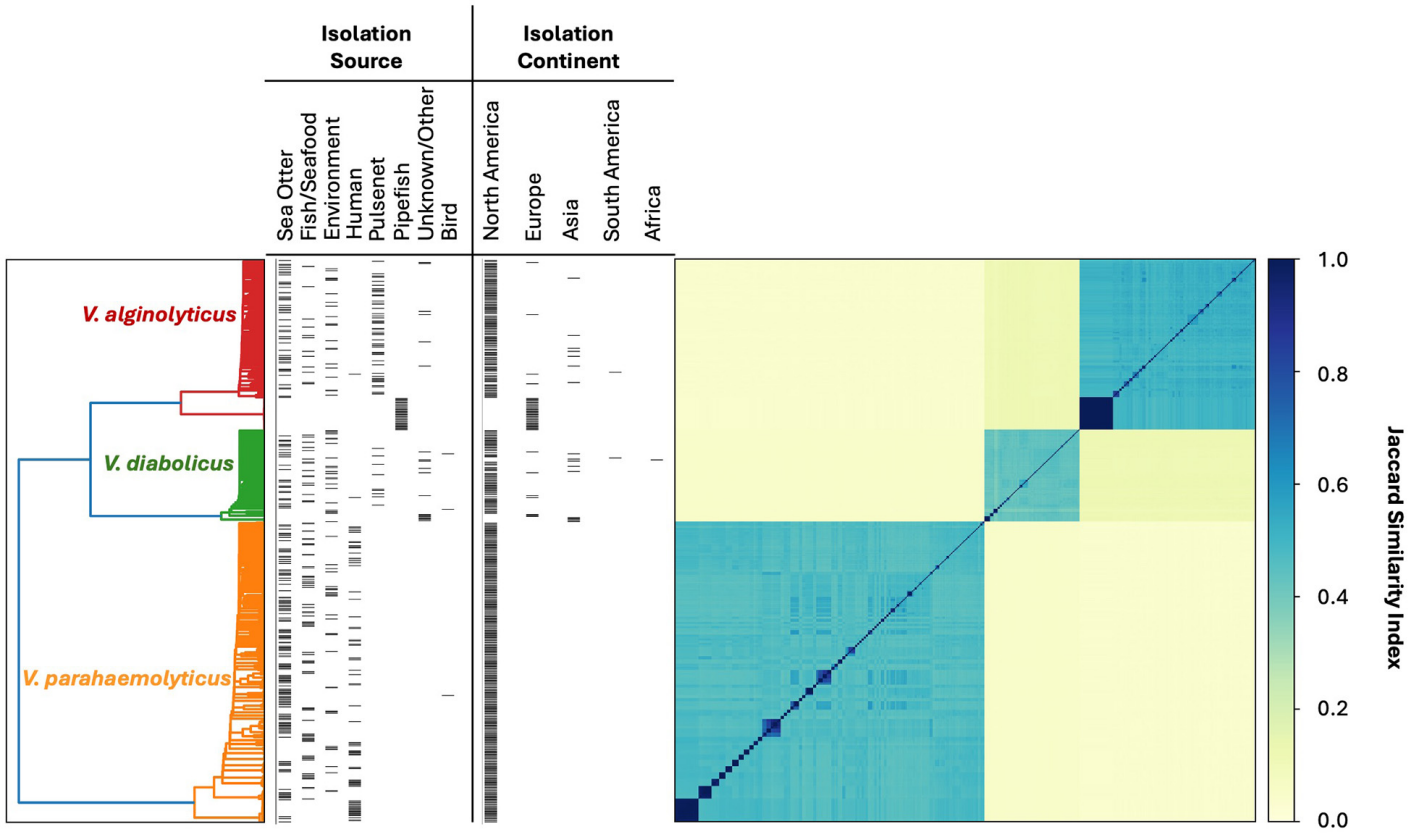
All-against-all Comparative analysis of the genomic similarity between *V. alginolyticus* (*n* = 163), *V. diabolicus* (*n* = 88), and *V. parahaemolyticus* (*n* = 287) genomes collected from various sources globally. Darker blue colors indicated greater similarity between genomes based on the Jaccard Similarity Index. All three species appropriately clustered into distinct non-overlapping regions of genomic similarity. The highest similarity between species was observed for *V. alginolyticus* and *V. diabolicus*. Colors on the non-rooted phylogenetic tree indicate species (*V. alginolyticus*; red, *V. diabolicus;* green, *V. parahaemolyticus;* orange). Genomes were labeled based on isolation source and continent where the isolate was obtained. Genomes where source was labeled as Pulsenet were from foodborne outbreaks and most likely from human cases.

After quality control screening, 36 *V. diabolicus* (one avian, eight environmental, four fish/seafood, 10 human, one cured hide, 12 unknown/other) and 108 *V. alginolyticus* (13 environmental, 9 fish/seafood, 49 human, 31 pipefish, six unknown/other) public genomes identified through Bracken were added to the 52 *V. diabolicus*, 55 *V*. alginolyticus, and 287 *V. parahaemolyticus* genomes examined previously (Supplemental Data 2). Whole genome phylogeny and similarity comparisons showed that all three species separated with 100% accuracy based on genomic identities, validating the re-assessed genomic species identities (Figure 2). Of the three species, *V. diabolicus* (*n* = 88) and *V. alginolyticus* (*n* = 163) shared the most genomic similarity, while *V. parahaemolyticus* and *V. alginolyticus* were the least similar. Isolates from humans and sea otters were represented across the genomic diversity of both *V. alginolyticus* and *V. diabolicus*. Other than the previously reported Kiel-Fjord ecotype of *V. alginolyticus* isolated from pipefish (*Syngnathus typhle*), there was limited evidence in *V. alginolyticus* of genomic clustering suggestive of minimal population structure. The next largest genomic cluster of *V. alginolyticus* included six genomes from the USA isolated from humans, southern sea otters, and estuarine water. Similarly, only small genomic clusters of *V. diabolicus* were observed, including five of the six Japanese isolates, and four of the USA estuarine water isolates sampled on the same day.

### 3.3 Pangenome and species-specific markers

**Figure 3:**
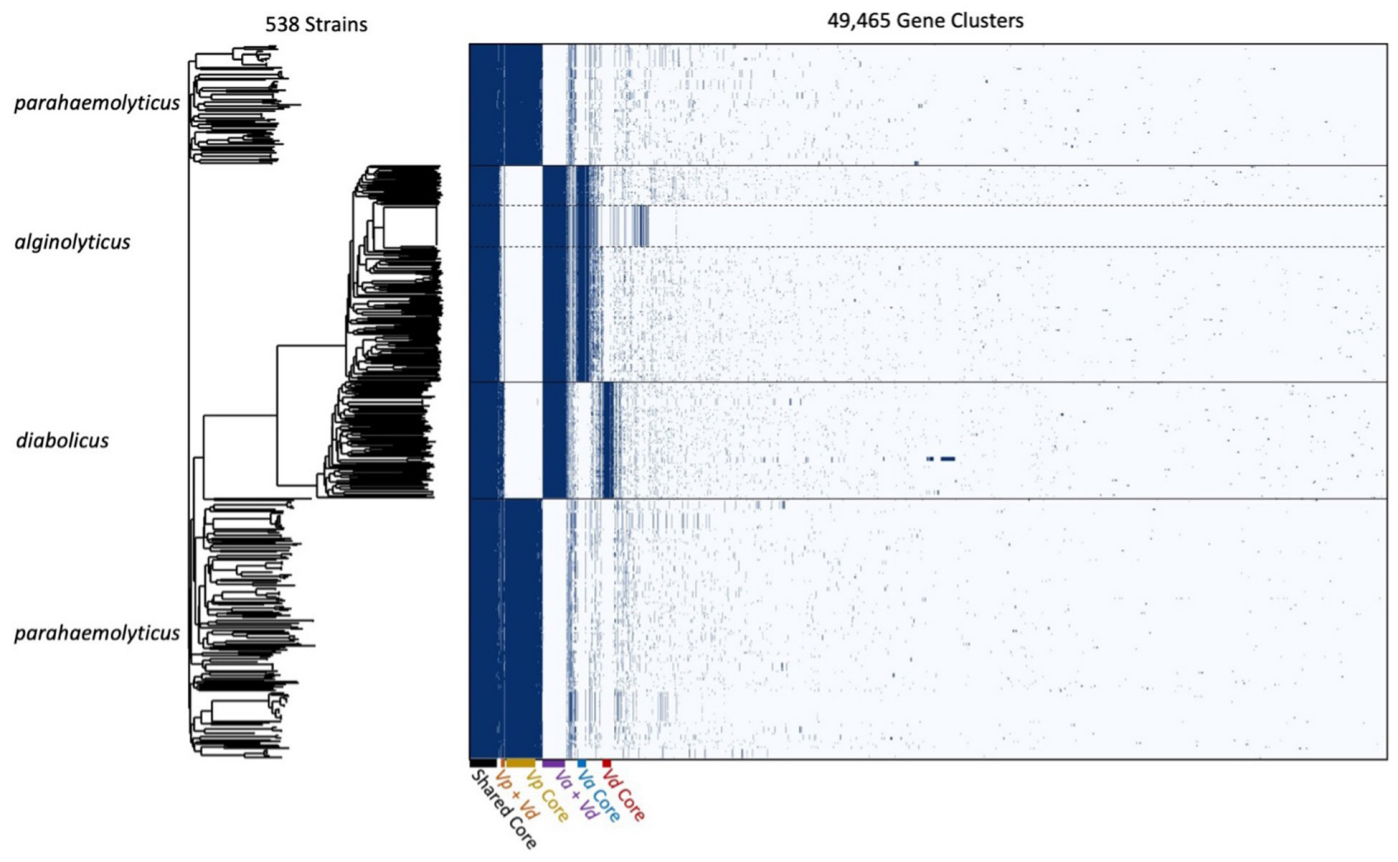
Combined pangenome for three closely related species *V. alginolyticus* (*n* = 163), *V. diabolicus* (*n* = 88), and *V. parahaemolyticus* (*n* = 287) collected from various sources globally. A 95% identity cutoff of the predicted amino acid sequences was used, which resulted in an extremely diverse gene pool (49,465 unique gene clusters). Species are separated on the heatmap of gene cluster presence by solid black lines while the Kiel-Fjord ecotype found in *V. alginolyticus* from pipefish are separated by dashed black lines. Core gene clusters shared across all species, two species, or individual species are color-coded at the bottom.

The shared pangenome of *V. alginolyticus, V. diabolicus*, and *V. parahaemolyticus* was extremely diverse with a comprised pool of 49,465 gene clusters. The combined pangenome analysis of all three species identified separate pangenome regions including a shared core genome, core genomes shared between 2 out of 3 species, and species-specific core genomes (Figure 3). Based on GWAS results, there were 643 gene clusters that highly associated with *V. alginolyticus* and 477 gene clusters highly associated with *V. diabolicus* (Supplemental Data 3 and 4). Many species-specific gene clusters were annotated as coding for hypothetical proteins despite utilizing two annotation tools. Previously reported antimicrobial resistance and virulence factor genes (Sebastian et al., 2025, 2026) were not present on species-specific gene cluster lists although *V. alginolyticus* and *V. diabolicus* included distinct allelic variants of putative antimicrobial resistance genes *mdtA* and *marR*.

**Figure 4:**
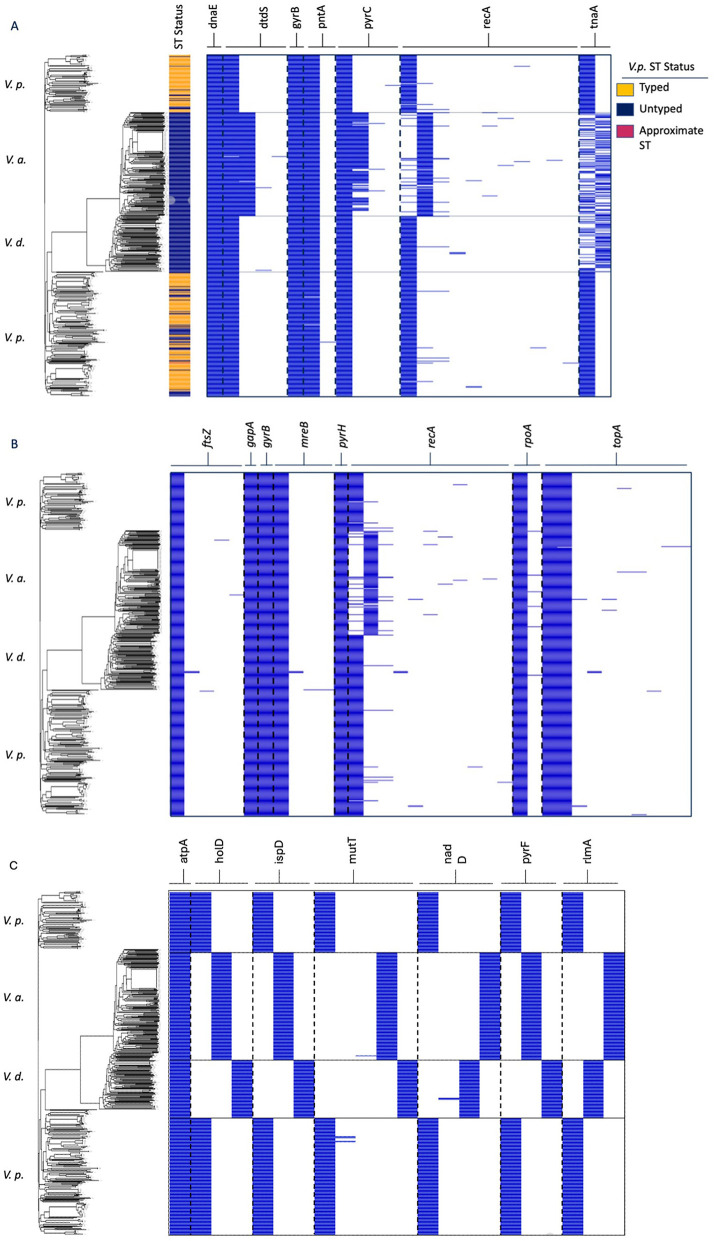
Heatmaps for presence (blue) or absence (white) of “housekeeping” genes used in MLST and MLSA schemes across *V. alginolyticus* (*n* = 163), *V. diabolicus* (*n* = 88), and *V. parahaemolyticus* (*n* = 287) genomes collected from various sources globally. **(A)** Heatmap for the gene clusters with annotations matching the seven loci *V. parahaemolyticus* MLST scheme (González-Escalona et al., 2008). **(B)** Heatmap for the gene clusters with annotations matching an 8 loci *Vibrio* spp. MLSA scheme (Sawabe et al., 2013). **(C)** Heatmap for atpA from the 4 loci *Vibrio* spp. MLSA scheme (Rahman et al., 2014) along with additional genes considered for future MLSA/MLST schemes. Genes were segregated into gene clusters using a 95% identity cutoff of predicted amino acid sequences. Dashed black lines separate gene loci while columns between dashed black lines compile all gene clusters with annotation to the labeled gene loci. Horizontal solid lines separate genomes by species. The heatmaps and genome phylogeny were visualized and reproduced using Phandango (Hadfield et al., 2018).

Presence or absence of genes annotated to gene names used in the seven loci *V. parahaemolyticus* MLST scheme or the eight loci MLSA scheme were plotted alongside the pangenome phylogeny (Figures 4A, B). The *atpA* gene from the four loci MLSA scheme, which overlaps three genes with the eight loci scheme, along with six additional putative “housekeeping” genes from the species gene cluster lists were plotted alongside the pangenome phylogeny (Figure 4C). Only five loci examined resulted in just one gene cluster shared between all strains across species (*dnaE, gyrB, gapA, pyrH, atpA*) signifying that any allelic divergence between species was present at < 5% variation.

Five of seven MLST and five of eight MLSA loci exhibited sequence divergence at a < 95% identity between variants, indicative of allelic variation. Sequence divergence of these loci resulted in gene clusters with < 100% species specificity. In some cases, multiple alleles with a shared gene annotation were present in the same genome. A genetic marker shared between the MLSA and MLST schemes, *recA*, had 11 annotated gene products with < 95% shared identity. One allele of *recA* was primarily restricted to *V. alginolyticus*, but was present in limited *V. diabolicus* and *V. parahaemolyticus* strains. The other loci with more than one annotated gene cluster were MLST genes *dtdS, pntA, pyrC, tnaA*, and MLSA genes *ftsZ, mreB, rpoA*, and *topA*. Six loci (*holD, ispD, pyrF, rlmA, mutT, nadD*) were selected through pan-GWAS filtering as putative markers for MLSA given integral functions in other bacterial species (Jensen et al., 1984; Carter et al., 1993; Lian et al., 2015; Jin et al., 2016; Rocha et al., 2016; Booth et al., 2017). Four loci exhibited exactly three gene clusters with 100% species specificity to *V. alginolyticus, V. diabolicus*, and *V. parahaemolyticus* while *mutT* and *nadD* carried two and one additional rare gene clusters, respectively.

All the *V. alginolyticus* and *V. diabolicus*, and 22.6% of the *V. parahaemolyticus* isolates were non-typeable using the *V. parahaemolyticus* MLST scheme. Most of the MLST loci (*dnaE, pntA, pyrC, tnaA*) could not be typed in any *V. alginolyticus* or *V. diabolicus* genomes, while *recA, dtdS*, and *gyrB* exhibited limited utility in both species. A majority of the untyped *V. parahaemolyticus* belonged to genomes from California of either environmental (*n* = 19/29, 65.5%) or southern sea otter sources (*n* = 39/106, 36.8%) (Sebastian et al., 2025, 2026).

### 3.4 Host-associated selection

Genomes of *V. alginolyticus* isolated from humans (*n* = 49) were either positively or negatively associated with 109 different gene clusters compared to *V. alginolyticus* from sea otters (*n* = 45, **Supplemental Data 5**). No *V. diabolicus* gene clusters significantly differed between human (*n* = 10) and sea otter (*n* = 25) hosts after Benjamini–Hochberg adjustment. Due to the lower sample size of *V. diabolicus* genomes, 36 gene clusters with significant non-adjusted lrt *p*-values and large effect sizes worthy of further investigation were reported (**Supplemental Data 6**). Many of the host-associated gene clusters were annotated as coding for hypothetical proteins. Few host-associated gene clusters were annotated to known or putative antimicrobial resistance genes. A multidrug resistance protein gene (*mdtk_1*) was associated with isolation from humans compared to sea otters (OR = 28.5 (2.66, 305.39), adjusted lrt *p* = 0.038). The antimicrobial resistance gene clusters annotated as chloramphenicol acetyltransferase (group_3651, *cat_1*; OR = 21.76 (0.76, 621.17), ltr *p* = 0.014, adjusted *p* = 1) and AmpC beta-lactamase (group_1644, *ampC*; OR = 8.5 (0.53, 127.44), ltr *p* = 0.046, adjusted *p* = 1) were included in **Supplemental Data 6** as pre-adjusted associations with *V. diabolicus* isolation from sea otters compared to humans isolates. The genes *ampC* and *cat_1* were directly next to each other in the genome. One of 25 genomes isolated from an otter was missing both *ampC* and *cat_1* completely compared to five of ten from humans. Two separate gene clusters were annotated to *cat_1* (groups 3651 and 3652) and a comparison of their predicted protein sequences revealed a mostly well conserved sequence, but with distinct substitutions and two group_3652 sequences that had additions likely to impact protein structure (**Supplemental Figure 1**). One genome isolated from a human had both group 3652 and 3651 clusters, but the gene likely underwent rearrangement resulting in two fragmented genes sequentially out of order. However, neither group 3652 nor group 3651 were associated with phenotypic chloramphenicol resistance in southern sea otter isolates, as all isolates were chloramphenicol susceptible. Predicted proteins of the *ampC* gene clusters exhibited allelic variation without a distinct pattern between gene clusters (**Supplemental Figure 2**). There was not enough phenotypic resistance data from previous minimum inhibitory concentration testing (Sebastian et al., 2025) to definitively link specific alleles with phenotypic ß-lactam resistance. Results that highlight the utility of GWAS to detect host-specific genes related to virulence or host adaptation are further described.

Multiple toxin-antitoxin (TA) systems, common in many bacteria species, exhibited a host preference for genomes from sea otters. Genes annotated to three distinct TA systems were highly associated with *V. alginolyticus* isolates from sea otters compared to *V. alginolyticus* isolates from humans (Table 1). A variants of cold shock protein V (*cspV*; group_19191) was strongly associated with *V. alginolyticus* from sea otters compared to humans (OR = 113.30 (2.16, 5938.43), adjusted lrt *p* < 0.001). Almost all *V. alginolyticus* genomes carried a variant of the putative virulence factor, *apx1B*, but different variants were associated with isolation from either sea otters or humans. A gene involved in biofilm formation as part of an amyloid secretion channel, *csgG* (Goyal et al., 2014), was associated with *V. alginolyticus* from human sources compared to sea otters (OR = 16.12 (2.56, 101.54), adjusted lrt *p* = 0.02). While *csgG* is characterized for *V. parahaemolyticus* (Karan et al., 2021), this *csgG* variant was nearly exclusive to *V. alginolyticus*. The two most frequently studied virulence factors of *V. parahaemolyticus*, the thermostable direct hemolysin (*tdh*) and thermostable direct hemolysin-like hemolysin (*trh*) (Raghunath, 2015) were not detected in *V. alginolyticus* and *V. diabolicus* genomes except for one *V. diabolicus* genome (SRR10099733) which carried *trh*.

**Table 1:** Toxin gene clusters from toxin-antitoxin (TA) systems in *V. alginolyticus* which were more frequently detected in sea otter compared to human isolation sources based on naïve lrt *p*-value.

Gene cluster	Odds ratio (95% CI)	lrt *p*-value	B–H adjust lrt *p*-value
*ccdB*	9.30 (1.10, 78.76)	0.032	n.s.
*higB*	42.10 (4.25, 417.05)	< 0.001	0.024^*^
*parE* group 1160	53.52 (5.19, 551.37)	< 0.001	0.013^*^
*parE* group 6999	7.03 (0.59, 83.06)	< 0.001	n.s.
*parE* group 13663	12.81 (2.25–72.86)	0.002	0.036^*^
*yoeB*	16.78 (2.61, 107.99)	0.002	0.040^*^

^*^denotes the gene clusters that were significantly associated with sea otters based on Benjamini–Hochberg adjustment of the lrt p-value.

## 4 Discussion

### 4.1 Implications of species identification inaccuracy in the Harveyi clade

Comparison of reported and re-classified species identities across 3,442 Harveyi clade genomes revealed that *V. diabolicus* has been systematically underreported. The misclassification of *V. diabolicus* genomes predominantly has been due to an inability to reliably separate *V. alginolyticus* and *V. diabolicus*, with reported *V. alginolyticus* being the least accurate species in the clade. Given the frequency of misidentification in SRA files labeled as *V. alginolyticus*, care should be taken in interpretation of prior studies that utilized public *V. alginolyticus* files without independent species verification.

The genomic based classification approach used here provides a quick and robust species resolution between three closely related species in the Harveyi clade (*V. alginolyticus, V. diabolicus*, and *V. parahaemolyticus*). Concordance between Bracken species identities and k-mer whole genome similarity clustering provided internal validation of the genomic reclassifications. This approach benefits from leveraging genome-wide sequences unlike MALDI-ToF or MLSA, which, because of limited data points to compare, have previously failed to discriminate *V. alginolyticus* and *V. diabolicus* (Rahman et al., 2014; Culot et al., 2021; Sebastian et al., 2025). The genomic similarity approach was successful even in distinguishing between species with average nucleotide identities (ANI) of ~92%, close to the 95% threshold for species delineation (Turner et al., 2018). Future genomic studies of *Vibrio* spp. should prioritize genome wide similarity or ANI over lower resolution methods to ensure accuracy of submitted species names in public databases.

Due to limitations in public domain SRA files, original species identification methods could not be confirmed, but multiple causes may explain the mismatches observed between reported species identification and genomic analysis identification. Many of the mismatches related to challenges in identifying closely related species. Frequent recommendations for taxonomic changes within the Harveyi clade have occurred in recent years, highlighting the need to revisit previously published genomes that have utilized outdated taxonomies (Hoffmann et al., 2012; Sawabe et al., 2013; Urbanczyk et al., 2013; Turner et al., 2018; Jiang et al., 2022). Data entry errors or mixed and contaminated sequencing may have explained a limited number of misclassifications. Within the NCBI SRA database, there were frequent discrepancies in how additional metadata fields were or were not utilized, leading to loss of data which could have been utilized by epidemiological and phylogenetic investigations. Evidence for mixed and contaminated sequencing included genomes with up to five ranked IDs and top ranked IDs outside of the *Vibrio* genus. Variations in sequencing depth or quality were also present, which may reduce confidence in species identifies.

Reclassification efforts increased the number of *V. diabolicus* genomes, including identification of 10 human clinical strains, expanding current evidence that *V. diabolicus* poses a public health threat. Recent isolations from varied seafood, including fish (Lucero-Olachea et al., 2026), shrimp (Cota-Ortega et al., 2026), and mussels (Yibar et al., 2025), demonstrate the need for a One Health approach to address *V. diabolicus* infections in humans and aquaculture. While the initial discoveries of *V. diabolicus* in 1991 (and synonymy with *V. antiquarius* in 1999) were from deep sea vents in the East Pacific rise region (Raguénès et al., 1997; Hasan et al., 2015), the addition of genomes across continents, isolation sources, and time periods suggest a global distribution. As additional *V. diabolicus* genomes continue to be published (Teixeira et al., 2022a,b,c; Yibar et al., 2025), application of ‘molecular clock' analyses could prove beneficial to reconstruct historical dissemination patterns *V. diabolicus* transmission waves as well as investigate evolutionary divergence from *V. alginolyticus* (Dos Reis et al., 2015).

### 4.2 Intraspecies diversity and genomic comparisons

Within each species, considerable strain diversity was observed, consistent with previous studies and the genomic plasticity characteristic of *Vibrio* species (Banerjee et al., 2018; Yang et al., 2019; Prithvisagar et al., 2021). The sample sizes of *V. alginolyticus* and *V. diabolicus* in this study, while representative of the most comprehensive genomic collection at the time, were insufficient to capture the full potential of species-wide diversity. The addition of new genomes is likely to contribute new gene content based on previously open pangenomes of both species (Sebastian et al., 2025, 2026). Studies wishing to capture the genetic breadth of these species should utilize the largest possible genome set from varied isolation sources, as clinical strains may represent only a fraction of overall intraspecies diversity (Yang et al., 2019).

The Kiel-Fjord ecotype of *V. alginolyticus* formed a prominent genomic cluster within the dataset (Chibani et al., 2020a,b), although additional evidence for genomic clustering of *V. alginolyticus* and *V. diabolicus* was limited. The next largest genomic clusters were comprised of six *V. alginolyticus* genomes and five *V. diabolicus* genomes. Increased sequencing of *V. alginolyticus* and *V. diabolicus* genomes may identify potential for clonal expansion, although it is critical that future studies provide thorough standardized metadata including collection date, isolation source, and geographic origin to maximize their utility.

Highly related genomes may also represent selection bias due to spatiotemporally localized sampling efforts. Previously, genomic similarity analysis of multiple isolates taken from the same host was used to remove redundant strains that may bias epidemiologic investigations (Sebastian et al., 2025, 2026). In this study, population structure was assessed through additional permutation analyses to ensure that gene presence was not phylogenetically biased. Even a weak correlation in population structure, as observed here, could impact downstream GWAS analyses and adjustment should be considered.

### 4.3 Applications of GWAS for species-specific markers

Genome-wide association studies of the combined pangenome of all three species identified gene clusters unique to or highly associated with *V. alginolyticus* (*n* = 643) and *V. diabolicus* (*n* = 477) indicative of divergent evolution between three closely related species. Many species-specific gene clusters encoded hypothetical proteins, highlighting the need for further characterization of *V. alginolyticus* and *V. diabolicus*. The high proportion of unannotated genes from GWAS provides targets for future functional characterization studies that are far more manageable than targeting all genes in the combined pangenome.

Distinct allelic variants of antimicrobial resistance genes *mdtA* and *marR* were present in both *V. alginolyticus* and *V. diabolicus* which were not previously detected using public antimicrobial resistance gene databases (Sebastian et al., 2025). Comprehensive Antibiotic Resistance Database (Jia et al., 2017; Alcock et al., 2022) and similar databases are built primarily for well-characterized pathogens, and still likely underestimate the resistance mechanisms of *V. alginolyticus* and *V. diabolicus*. The GWAS framework applied here can complement database searches in two key ways: (1) to start with a shortened list of target genes for experimental validation which can greatly improve study power and/or reduce needed sample sizes and (2) similar GWAS methods can be applied with future phenotypic data, such as minimum inhibitory concentration testing, when available to discover allelic variants associated with AMR.

Species-specific gene clusters were also applied to detection of markers for improved species identification schemes, although future studies have the potential to identify additional markers for MLSA/MLST by further interrogating the species-specific gene cluster lists provided. Putative “housekeeping” genes *holD, ispD, pyrF*, and *rlmA* all exhibited species-specific gene clusters with 100% accuracy of species classification within the current dataset and could be incorporated in future studies to experimentally validate novel MLSA schemes. In contrast, commonly utilized MLST and MLSA schemes include multiple markers that exhibited allelic variation with greater than 5% sequence divergence and sometimes carried more than one gene cluster annotated to the same “housekeeping” gene. The *recA* gene (González-Escalona et al., 2008; Culot et al., 2021) is a prime example of a commonly used MLSA/MLST loci despite concerning allelic variation (Figure 4) and susceptibility to frequent recombination events that can affect its usefulness for species identification and typing (Gunasekara et al., 2023).

While whole genome methods are capable of the finest species resolution, lower resolution methods including MLSA remain popular because they require a limited number of loci and can be analyzed with potentially lowered costs (Jiang et al., 2022). Validating novel MLSA schemes with the putative loci identified here will include additional challenges in finding appropriate primer sites in allelic variants to ensure PCR amplification across *Vibrio* spp. and determination of whether allelic variation patterns extrapolate to consistent segregation of other *Vibrio* spp. An approach using core genome MLST (cgMLST) for species identification also has merit, although would not solve the need for species identification and typing in labs that lack whole genome sequencing capacity. Since no validated cgMLST scheme exists for *V. alginolyticus* and *V. diabolicus* and our results show significant differences in core genes that would make *V. parahaemolyticus* cgMLST ineffective, it is most logical to instead utilize the whole genome species classification and similarity clustering as presented here.

### 4.4 Applications of GWAS for host-associated markers

Genome-wide associations were successfully utilized to identify gene clusters associated with either human or sea otter isolation sources. Because of the limited population structure observed in relatively diverse species and the use of a population structure adjustment, the identified gene clusters were most likely due to true host-specific niche adaptation instead of phylogenetic confounding. However, ecological or exposure differences between strains could not be ruled out. The previously described Kiel-Fjord ecotype provides an extreme example of niche adaptation, with selection of a very specific *V. alginolyticus* genomic profile in pipefish (Chibani et al., 2020a,b). Notable but less extreme putative examples of novel host adaptations in *V. alginolyticus* and *V. diabolicus* were detected, including gene-host associations relating to antimicrobial resistance, virulence, and biofilm formation.

Toxin-Antitoxin (TA) system gene clusters were associated with isolation in sea otter hosts compared to human hosts, including variants of *higB, parE*, and *yoeB*. The TA systems have major implications for pathogenicity as exhibited by an association of the *V. parahaemolyticus* virulence gene *pirA*, linked to acute hepatopancreatic necrosis disease (AHPND) in shrimp, and the TA system gene *CcdB* (Vandeputte et al., 2024). TA systems have diverse functions in *Vibrio* spp., which include maintenance of a viable but non-culturable state, maintenance of genomic and plasmid DNA, protection from phages, colonization, and biofilm formation (Budde et al., 2007; De Jonge et al., 2010; Zheng et al., 2015; Song et al., 2022). TA system genes are likely shared via horizontal gene transfer, as evidenced by an environmental strain of *V. diabolicus* (JBS-8-11-1) carrying a fitness island with 13 TA system genes (Klein et al., 2018). A similar fitness island allowing for *V. alginolyticus* or *V. diabolicus* to better compete within marine mammal hosts might explain the presence of sea otter-associated TA systems, but requires further functional experimentation.

An allelic variant of cold shock protein V (*cspV*) was also associated with sea otter hosts in *V. alginolyticus*. Although cold shock proteins (csp) are frequently considered part of the core genome critical for to survival in harsh conditions (Datta and Bhadra, 2003; Zhu et al., 2017; Nam et al., 2022), non-core *csp* genes may confer functions beyond thermal stress response. In *Vibri*o spp., *csp* genes can have additional functions of regulation of biofilm formation and expression of Type VI Secretion Systems (Datta and Bhadra, 2003; Townsley et al., 2016; Nam et al., 2022). While speculative, sea otter host associated *cspV* of *V. alginolyticus* could have evolved to face distinct environmental conditions not necessary for human infection such as improved gut colonization in cold marine waters.

The *apx1B* gene, involved in repeat-in-toxin (rtx) translocation as part of the Type I Secretion System (T1SS) (Linhartová et al., 2010; Benz, 2016; Cho and Yoon, 2021), was present in nearly all *V. alginolyticus* genomes, but with a specific gene cluster associated with isolation from either sea otter or human hosts. Host-associated allelic variation of this gene could indicate that Rtx toxin secretion is host-adapted to improve gut colonization to different host epithelial cells. Association with human hosts was observed for a *csgG* allele of the CsgF-CsgG protein complex, which functions as a secretion channel for curli fibers and promotes biofilm formation (Goyal et al., 2014; Yan et al., 2020). While characterized primarily in *V. parahaemolyticus* (Karan et al., 2021), the *csgG* cluster detected was nearly exclusive to *V. alginolyticus*, suggesting both species-specific and host-specific adaptations to biofilm regulation.

No gene associations remained significant in *V. diabolicus* after both population structure and Benjamini–Hochberg corrections, reflective of the limited statistical power given the sample size. Gene clusters potentially associated with either sea otters or humans prior to statistical correction presented are noteworthy but should be interpreted cautiously, such as the putative associations of antimicrobial resistance genes chloramphenicol acetyltransferase (*cat_1*) or beta-lactamase (*ampC*) with sea otter isolation. Both genes were positioned next to each other and thus horizontal gene transfer could confer multiple antibiotic resistance. Allelic variation was observed that putatively would alter protein functions, and functional experimentation should characterize the resistance potential of variants of both genes. Previously published antimicrobial resistance phenotypes in a subset of *V. diabolicus* genomes (*n* = 18) isolated from Southern sea otters (Sebastian et al., 2025) were not associated with specific allelic variants of *cat_1* or *ampC*, although future experimental studies could examine the impact of single nucleotide polymorphisms identified here (Supplemental Figures 1, 2) on gene function. Antimicrobial resistance and virulence mechanisms of *V. alginolyticus* and *V. diabolicus* are understudied and rely heavily on knowledge from *V. cholerae* and *V. parahaemolyticus*, especially for annotated genes in antimicrobial resistance and virulence databases (Xie et al., 2005; Ren et al., 2013; Xiong et al., 2014; Hernández-Robles et al., 2016; Pérez-Duque et al., 2021; Cai et al., 2022). A genome-wide association study approach, as used in this study, can refine searches for novel or understudied virulence genes and gene variants agnostic to what is reported in better characterized species. Additional applications of GWAS include detection of microbial genes associated with the presence of antimicrobial resistance phenotypes (Coll et al., 2022), virulence factors (Vandeputte et al., 2024), biofilm formation (Redfern et al., 2021), and disease severity (Bandoy and Weimer, 2020).

### 4.5 Limitations

Key limitations should be considered when interpreting these results. The GWAS approaches used provides associations, which can generate hypotheses for future studies. Causation cannot be proven through association and experimental validation through gene knockout or phenotypic characterization is recommended. Limited phenotypic data was utilized in this study given the lack of metadata relating to phenotype in public domain genomes. Metadata relating to antimicrobial susceptibility testing, virulence assays, or host-infection experiments should be linked to deposited genomes whenever possible to enhance future GWAS comparisons.

Another major limitation of the GWAS tools utilized is the use of repeated binomial tests, which require *post hoc* statistical correction to control for false discoveries. The dichotomous comparisons of many GWAS tools limit the ability to analyze non-dichotomous data such as multiple host types or other potential covariates such as geographic origin and collection date. While confounding from geographic origin biases was partially mitigated by the predominance of North American strains in the dataset, future studies from diverse geographic sources are needed to incorporate stratified analyses to better account for localized variation in *V. alginolyticus* and *V. diabolicus* strains.

Control for false discoveries over thousands of gene clusters requires a robust sample size for statistical power. Sample sizes may also limit the utilization of GWAS with effective studies potentially requiring hundreds if not thousands of genomes that are not available for emerging species such as *V. diabolicus* (Coll et al., 2022). The dataset provided here utilized all available SRA genomes of Harveyi clade genomes, although newer genomes continue to be published adding to the future viability of GWAS in emerging species. Our approach allowed for population structure correction, although given the sample size, much of the strain diversity of *V. alginolyticus* and *V. diabolicus* has not yet been characterized.

The use of publicly available genomes has inherent limitations in the inconsistency and reliability of metadata. The assumption that PulseNet genomes were human-derived fit with the program's stated scope and recorded “clinical” type isolate status, although even a few misclassified genomes could have impacted the results. Metadata standardization in public repositories is a critical need for comparative genomics. Despite the limitations addressed, the GWAS framework presented here was designed to generate targeted hypotheses around novel associations between *Vibrio* spp. and host-adaptation with the benefit to future studies of greatly reducing the number of candidate genes for mechanistic analyses.

## 5 Conclusions

This study confirmed that *V. diabolicus* genomes have been frequently misclassified as *V. alginolyticus* in public databases, and correcting these misclassifications substantially expands upon the limited epidemiologic and ecologic interpretations of an overlooked, emerging human and wildlife pathogen. The genomic methods including genome-wide similarity utilized here provided accurate and reproducible species identification which we recommend for future Harveyi clade categorization. For laboratories reliant on MLSA/MLST schemes as a quick and low-cost method, we identified currently used loci that may lead to a failure to appropriately classify strains as well as four candidate genes (*holD, ispD, pyrF*, and *rlmA*) that warrant future validation as improved MLSA markers capable of distinguishing the closely related species of *V. alginolyticus* and *V. diabolicus*, and *V. parahaemolyticus*.

Genome-wide association studies revealed host-specific gene associations in *V. alginolyticus*, including genes related to antimicrobial resistance, toxin-antitoxin systems, cold-shock, RTX toxin secretion, and biofilm formation. Although non-significant after population structure correction and false discovery adjustement, *V. diabolicus* antimicrobial resistance gene variants of *cat_1* and *ampC* were discovered as worthy targets for future phenotypic comparisons.

Due to predicted increases in *Vibrio* spp. infection from changing climates (Goertz et al., 2013; Baker-Austin et al., 2017), accurate identification of species and classification of high-risk strains is needed for One Health strategies to advance human and marine wildlife health. The GWAS framework demonstrated here provides a replicable pipeline for accurate identification and identification of candidate virulence, AMR, and host-adaptation genes in understudied species like *V. diabolicus*.

## Data Availability

Whole genome sequence reads (SRA files) with corresponding metadata have been made available on NCBI with BioSample numbers (SAMN40178385–SAMN40178873) as part of the 100K Pathogen Genome Project and initially published as part of a separate study (Sebastian et al., 2025). Additional previously published whole genome sequence reads (SRA files) were included in genomic analyses (Miller et al., 2021). All additional SRA files included in genomic analyses were downloaded from publicly available genomes on NCBI and listed in supplemental materials.
